# Cochlear Implantation in Children with Inner Ear Malformations: Auditory Outcomes, Safety and the Role of Anatomical Severity

**DOI:** 10.3390/jcm14228245

**Published:** 2025-11-20

**Authors:** Miriam González-García, Cristina Alonso-González, Francisco Ropero-Romero, Estefanía Berrocal-Postigo, Francisco Javier Aguilar-Vera, Concepción Gago-Torres, Leyre Andrés-Ustárroz, Manuel Lazo-Maestre, M. Amparo Callejón-Leblic, Serafín Sánchez-Gómez

**Affiliations:** 1 Otorhinolaryngology Service, Virgen Macarena University Hospital, 41009 Seville, Spain; cristinaalonso81gonzalez@gmail.com (C.A.-G.); roperoromero@gmail.com (F.R.-R.); estebepo@gmail.com (E.B.-P.); franagui2@hotmail.com (F.J.A.-V.); concheta08@gmail.com (C.G.-T.); leyre.andres.sspa@juntadeandalucia.es (L.A.-U.); manuel.lazo@faigesco.es (M.L.-M.); mcallejon@us.es (M.A.C.-L.); serafin.sanchez.sspa@juntadeandalucia.es (S.S.-G.); 2 Biomedical Engineering Group, University of Seville, 41092 Seville, Spain

**Keywords:** cochlear implant, hearing loss, inner ear malformations, INCAV classification, severity score, auditory outcomes, pediatric otology

## Abstract

**Background/Objectives**: Cochlear implantation (CI) has been shown to be effective in children with inner ear malformations (IEMs). However, outcomes vary with malformation type and anatomical complexity. Advances in radiological classification may improve the understanding of such variability to better guide patient counseling. We aimed to assess one-year post-implant auditory outcomes in children with IEMs using radiology-based classifications, and to explore genetic and perinatal predictors. We also propose a preliminary severity score derived from the INCAV system. **Methods**: Out of 303 pediatric CI recipients assessed at a tertiary center, we retrospectively analyzed 41 children (82 ears) diagnosed with IEMs. Malformations were categorized with the Sennaroğlu system and re-coded using INCAV, from which a severity score was derived. Postoperative outcomes were assessed in 56 implanted ears, including pure-tone average (PTA), word recognition score (WRS), and post-surgical complications. Statistical analyses included Spearman’s correlation, linear regression, and exploratory discriminant MANOVA. **Results**: The most frequent malformation was enlarged vestibular aqueduct (33%), followed by incomplete partition type II (22%). CI was performed in 56 malformed ears with a complication rate of 10.7%. PTA and WRS correlated with the INCAV-derived severity score, with higher severity linked to poorer thresholds and lower WRS. Linear regression showed severity explained ~20% of PTA variance, with outcomes more frequently impaired in ears with scores > 3. Exploratory analysis revealed inter-subject variability, with partial separation of mild versus moderate/severe groups mainly driven by PTA and WRS. **Conclusions**: CI in pediatric IEMs is safe and consistently improves hearing thresholds. PTA was the most robust predictor of performance, while the INCAV-derived severity score, though exploratory, may provide additional value for anatomical stratification, prognostic counseling, and rehabilitation planning.

## 1. Introduction

Inner ear malformations (IEMs) account for up to 20% of congenital sensorineural hearing loss and arise during early embryogenesis [1,2]. They represent a significant challenge for cochlear implantation (CI), as outcomes vary widely depending on malformation type, surgical feasibility, and cochlear nerve integrity. Careful preoperative imaging and classification are therefore essential for surgical planning and for setting realistic expectations with families [3].

Several classification systems have been proposed to categorize IEM. Jackler’s classification [4], based on embryogenesis, distinguishes five groups: complete labyrinthine aplasia or Michel deformity, cochlear aplasia, common cavity, cochlear hypoplasia, and incomplete partition. In 2002, Sennaroğlu and Saatci [1] introduced a new classification focused on anatomical cochlear differences in the cochlea, subdividing incomplete partition (IP) into two additional categories. In 2017, Sennaroğlu and Bajin [5] expanded the classification to eight types of cochlear malformations, also taking cochlear nerve anomalies into account. Another relevant classification is the INCAV system, proposed by Adibelli et al. in 2017 [6], which is radiologically based and aims to standardize the description of IEM, reduce subjective interpretation of radiologic imaging, define previously unclassified malformations, and aid in selecting CI candidates.

The prevalence of IEMs varies across populations, with an estimated incidence between 3.7% and 11.4% among children with congenital sensorineural hearing loss [2,7,8,9]. The most common anomalies are enlarged vestibular aqueduct (EVA) and incomplete partition type II (IP-II), which generally yield better auditory outcomes after CI, whereas more complex malformations, such as incomplete partition type I or common cavity, are typically associated with poorer results [2,7,8]. Beyond anatomy, syndromic conditions and genetic variants, such as SLC26A4 mutations in EVA or CHARGE syndrome, as well as congenital infections including cytomegalovirus and Zika virus, may further compromise cochlear development and negatively impact implant performance [10].

Functional outcomes after CI in children are often evaluated with structured auditory performance scales such as the Categories of Auditory Performance (CAP) or the Nottingham Auditory Milestones (NAMES). However, considerable heterogeneity remains across studies due to different test batteries applied, limiting the comparability of results and the ability to establish standardized benchmarks [11,12].

The aim of this study was to evaluate auditory outcomes one year after CI in children with IEMs, using both established and novel radiological classifications. In addition, we propose and preliminarily assess an exploratory severity score derived from the INCAV system as a potential tool to stratify anatomical complexity and refine prognostic counseling.

## 2. Materials and Methods

### 2.1. Study Design and Participants

We conducted a retrospective case series of patients under 18 years with severe to profound sensorineural hearing loss and radiologically confirmed IEMs who underwent cochlear implantation at Virgen Macarena University Hospital (Seville, Spain) between January 2008 and April 2025. The study followed STROBE recommendations for observational research.

Ethical approval was obtained in August 2024 and finalized in October 2024, covering the retrospective analysis of all data under institutional consent for research and educational purposes. No new patients were enrolled or intervened upon after the approval date; only follow-up information from the same previously treated cohort was incorporated. The study was approved by the local ethics committee (IC-MOI, Seville, Spain).

### 2.2. Classification of IEMs

All patients underwent temporal bone CT and MRI. IEMs were classified using two frameworks. First, the INCAV system [6] was applied, assigning each ear a unique five-domain code (internal auditory canal, cochlear nerve, cochlea, vestibular aqueduct, vestibule). During data analysis, we encountered difficulties with conventional classifications, since many ears displayed more than one malformation, and cases were duplicated across subgroups. To address this limitation, we developed an exploratory severity score derived from INCAV by summing ordinal values (0–3) for each domain to yield a global index (range 0–13). This approach avoided duplication, captured overall anatomical complexity, and reflected our observation that ears with higher values in individual INCAV domains tended to show poorer outcomes. For interpretability, ears were stratified as mild (1–3), moderate (4–7), or severe (≥8). Full coding rules and examples are provided in Supplementary Methods (Supplementary Table S1).

Second, malformations were also categorized according to the Sennaroğlu classification [5] to ensure comparability with previous reports, although this framework allows multiple anomalies to be assigned to the same ear.

### 2.3. Data and Outcome Measures

Clinical and demographic data included sex, age at implantation, onset of hearing loss (pre- vs. postlingual), implant side, time from diagnosis to implantation, stimulation mode (unilateral, bilateral simultaneous, bilateral sequential, or bimodal), implant model, number of procedures, and perinatal risk factors (prematurity, hyperbilirubinemia, low birth weight, meningitis, Specific Language Impairment (SLI), Autism Spectrum Disorder (ASD), Cytomegalovirus (CMV) infection, and genetic disorders). In addition, information on associated comorbidities was recorded when available, including neurodevelopmental and systemic disorders such as epilepsy, spinocerebellar ataxia, Down syndrome, or CHARGE syndrome, which may represent additional burdening factors influencing auditory outcomes.

Audiological outcomes were measured one year after implantation. All tests were conducted in sound-treated, silent booths under controlled acoustic conditions. Pure-tone averages (PTA) were obtained at 500, 1000, 2000, and 4000 Hz. Speech perception was assessed using open-set recorded Spanish disyllabic and trisyllabic word lists HL (Marrero-Cárdenas, 1994) [13], presented in free field at 65 dB HL and without lip-reading whenever possible, particularly in postlingually implanted children. To facilitate participation, some children were supported with visual aids such as color cards, though this approach remained exploratory. In bimodal users, the non-implanted ear was masked using speech-shaped noise during speech testing and narrow-band noise during pure-tone audiometry, as appropriate to prevent cross-hearing. For prelingually implanted or very young children unable to complete word recognition testing, Auditory Brainstem Responses to speech stimuli (ABRIS) were used to objectively assess auditory perception. In these cases, results were documented as “no response”, and when applicable, a conservative score of 0% was assigned for statistical analysis.

Functional development was assessed with two validated tools: the NAMES scale [12], which tracks early auditory behaviors in five stages, and the CAP scale [11], an eight-level hierarchy from no sound awareness to telephone use. Scores were extracted retrospectively from speech therapy records.

### 2.4. Data Handling and Missing Values

Postoperative data were missing in 7% of ears for PTA (*n* = 4), 27% for WRS (*n* = 16), and >70% for CAP and NAMES. Missing PTA values were imputed with the mean, given the low proportion and symmetric distribution. For WRS, two scenarios were distinguished: in prelingual children or too young to reliably perform speech discrimination, results were documented in medical records as “no response” and conservatively coded as 0%; when no testing was performed or no data were available, values were left as missing. CAP and NAMES were excluded from analyses due to the high rate of missingness.

### 2.5. Statistical Analysis

Analyses were performed in SPSS v27 and MATLAB v2024b. Descriptive statistics were first used to summarize demographic, clinical, anatomical, and surgical variables. Continuous data are reported as medians and interquartile ranges (IQR), while categorical variables are presented as counts and percentages. Normality was tested with the Shapiro–Wilk test. Pairwise associations between severity, outcomes, and risk factors were examined with Spearman’s ρ (95% bootstrap CI). Linear regression explored relationships between severity and PTA/WRS. Canonical discriminant analysis (MANOVA) was applied to assess whether combined profiles (PTA, WRS, sex, CI type) could discriminate between mild and moderate/severe groups. All tests were two-tailed with α = 0.05.

## 3. Results

### 3.1. Patient Characteristics

**Figure 1:**
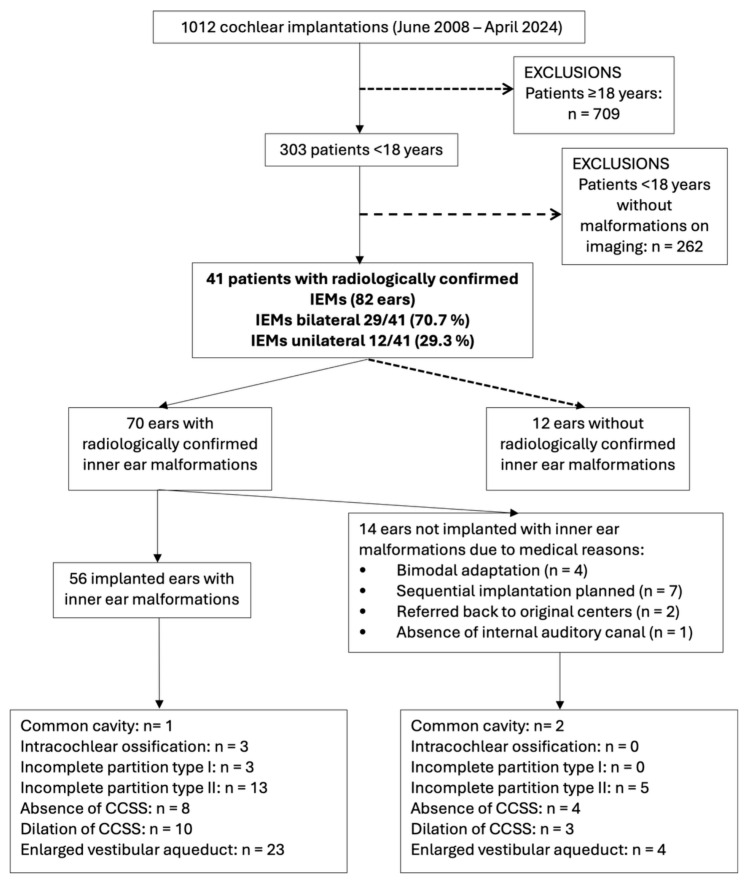
This flowchart summarizes the selection of pediatric cochlear implant recipients with inner ear malformations. Final inclusion was limited to 56 implanted ears with confirmed malformations. Malformations were classified using the mutually exclusive INCAV coding system to avoid duplication in subgroup analysis. Abbreviations: IEMs (Inner ear malformations).

A total of 1012 patients underwent cochlear implantation between January 2008 and April 2025 at the Virgen Macarena University Hospital in Seville, Spain. Of these, 303 were younger than 18 years at the time of surgery. Forty-one pediatric patients (13.5% of the under-18 cohort) had radiologically confirmed inner ear malformations (IEMs), corresponding to 82 ears analyzed descriptively. Postoperative auditory outcome analyses were restricted to the 56 ears that underwent cochlear implantation, while the remaining 26 ears were either anatomically normal (*n* = 12) or were not implanted due to surgical contraindications or planned sequential strategies (*n* = 14). Figure 1 summarizes the patient and ear flow through the study.

All patients were assessed one year post-implantation. The median follow-up period was 65.5 months (IQR, 42–103.5). Device activation occurred within 2–3 weeks after surgery in all cases. The implanted cohort included 29 males (70.7%) and 12 females (29.3%), with ages at implantation ranging from 1 to 17 years (mean 5.3 years, median 5 years). Regarding auditory stimulation mode, sequential bilateral implantation was the most frequent approach (34.1%, *n* = 14), followed by unilateral implantation (26.8%, *n* = 11). Both bimodal adaptation and simultaneous bilateral implantation were observed in 19.5% of patients (*n* = 8 each).

Hearing loss was classified as prelingual in 19 patients (46%) and postlingual in 22 (54%). The median age at implantation was 4 years, with most cases (61.0%) occurring between 3 and 10 years. A total of 19 patients (46.3%) were implanted at or before 3 years of age, and 5 patients (12.2%) between 11 and 17 years. The age distribution was positively skewed (skewness = 1.33), reflecting a predominance of younger implantation ages.

### 3.2. Inner Ear Malformations (Sennaroğlu Classification)

According to the Sennaroğlu system, the most common malformation was enlarged vestibular aqueduct (EVA), present in 27 of 82 ears (32.9%), followed by incomplete partition type II (IP-II) in 18 ears (21.9%). Semicircular canal abnormalities were also frequent, including dilatation in 13 ears (15.9%) and absence in 12 (14.6%). Less common anomalies included incomplete partition type I, common cavity, and intracochlear ossification (3 ears each, 3.7%).

Detailed distributions are shown in Table 1. Postoperative PTA and WRS values for each subtype are provided in Supplementary Table S2, but should be interpreted with caution, given the small sample sizes in several categories.

### 3.3. Device and Electrode Selection

Given the heterogeneity of inner ear malformations, device and electrode selection were adapted to each cochlea’s anatomy. A total of 56 malformed ears were implanted using 14 electrode models. The most frequent arrays were the Synchrony 2 MI1250 Flex 28 (MED-EL, Innsbruck, Austria, 17.9%) and CI632 Slim Modiolar (Cochlear, Sídney, Australia, 14.3%), followed by the AB Ultra 3D Mid-Scala (Advanced Bionics, Stäfa, Suiza) and CI512 Contour Advance (Cochlear, Sídney, Australia), each used in 10.7% of cases.

Electrode choice followed an anatomically guided rationale: shorter or modified arrays (e.g., Form 19, Form 24, Contour Advance, Cochlear, Sídney, Australia) were used for severe malformations such as incomplete partition I, common cavity, or ossification, while straight or mid-scala electrodes (e.g., Flex 28, Slim Modiolar, MED-EL, Innsbruck, Austria) were preferred for incomplete partition II.

Intraoperative radiographic verification was routinely performed in complex cases, and postoperative imaging was obtained when clinically indicated. Representative examples are shown in Supplementary Figure S1, and the detailed distribution of implant models is summarized in Supplementary Table S3.

### 3.4. INCAV-Derived Severity Score Distribution

Each ear received a unique INCAV code, and a cumulative severity score (0–13) was calculated. Among the 82 ears, most malformations were mild (*n* = 60; 73.2%), usually representing isolated vestibular defects such as EVA or semicircular canal abnormalities. Moderate-to-severe malformations accounted for 10 ears (12.2%), including IP-I, common cavity, and cochlear nerve hypoplasia/aplasia. Twelve ears (14.6%) had normal anatomy and were not classified as IEMs.

This severity-based stratification was subsequently used to assess auditory outcomes and implantability (Table 2). A complete description of the coding system and point allocation is provided in Supplementary Tables S1 and S4.

### 3.5. Associated Risk Factors

Perinatal and neonatal risk factors commonly linked to hearing loss were identified in the cohort (Table 3). SLI was observed in 29.3% of cases, genetic alterations in 21.9%, NICU stay in 21.9%, low birth weight in 9.8%, CMV infection in 7.3%, and prematurity in 5.0%. Less frequent factors included meningitis (3.7%) and hyperbilirubinemia (1.2%).

The most frequent genetic findings were SLC26A4 mutations and CHARGE syndrome (*n* = 4 each, 4.9%). Other alterations included compound GJB2/GJB6 mutations, DIABLO gene variants, and Down syndrome (*n* = 2 each, 2.4%). Additionally, some of these findings, such as CHARGE and Down syndromes, as well as cases with epilepsy or spinocerebellar ataxia, represent neurodevelopmental or systemic comorbidities that may influence auditory outcomes. Full details are shown in Table 4.

### 3.6. Postoperative Complications

Most implanted ears (89.3%) had no postoperative complications. Six events were recorded: gusher (*n* = 2; 3.6%), reimplantation (*n* = 2; 3.6%), subperiosteal abscess (*n* = 1; 1.8%), and transient peripheral facial paralysis (*n* = 1; 1.8%). All cases were successfully managed without permanent sequelae (Table 5).

### 3.7. Surgical and Postoperative Data

#### 3.7.1. Correlation Analyses

**Figure 2:**
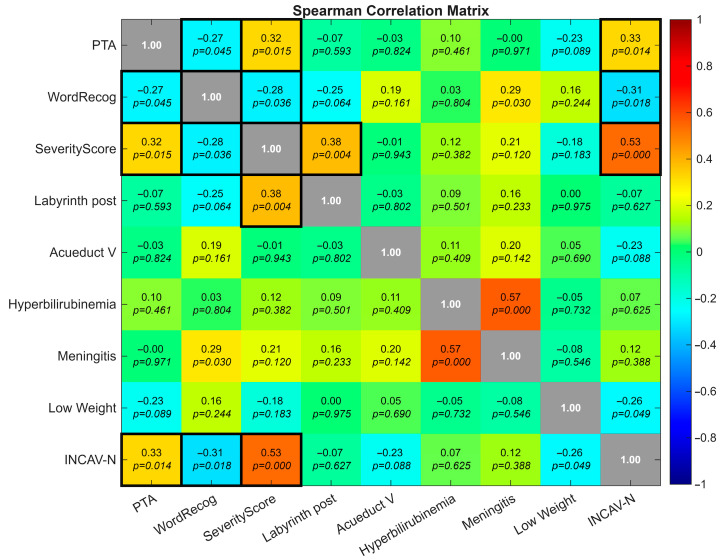
Heatmap showing pairwise correlations among auditory outcomes (PTA, WRS), severity score, anatomical subcomponents, and perinatal risk factors. Notable associations include inverse PTA–WRS correlation (ρ = −0.27, *p* = 0.045), positive PTA–severity score correlation (ρ = +0.32, *p* = 0.015), and cochlear nerve deficiency (INCAV-N) correlating with higher PTA, lower WRS, and greater severity (ρ = +0.33, −0.31, +0.53, respectively, with *p*-values 0.014, 0.018 and <0.01). Black boxes on the diagonal represent self-correlation values (ρ = 1.00).

Spearman’s rank coefficients revealed significant associations between anatomical severity and auditory outcomes (Figure 2). PTA and WRS were inversely correlated (ρ = −0.27, *p* = 0.045), confirming that poorer hearing thresholds were linked to reduced speech recognition. The severity score correlated positively with PTA (ρ = +0.32, *p* = 0.015) and negatively with WRS (ρ = −0.28, *p* = 0.036), indicating that greater anatomical complexity was associated with worse auditory performance. Cochlear nerve deficiency (INCAV-N) reinforced this pattern, correlating with higher PTA, lower WRS, and higher severity scores (ρ = +0.33, −0.31, +0.53, respectively, with *p*-values 0.014, 0.018, and <0.01). Posterior labyrinth malformations correlated positively with the Severity Score (ρ = +0.38, *p* = 0.004), indicating their association with more complex inner ear dysmorphogenetic. Perinatal and neonatal risk factors showed only weak or inconsistent correlations.

#### 3.7.2. Regression Analyses

Before performing MANOVA and linear regression, the requisite assumptions were tested. Although the Lilliefors test indicated significant deviations from normality (*p* < 0.05), Q–Q plots revealed that these departures were mild and attributable to highly quantized variables. Homogeneity of variance was confirmed by Levene’s test (*p* > 0.05 for all groups), and Variance Inflation Factors (VIF = 1.11–1.41) indicated negligible multicollinearity.

**Figure 3:**
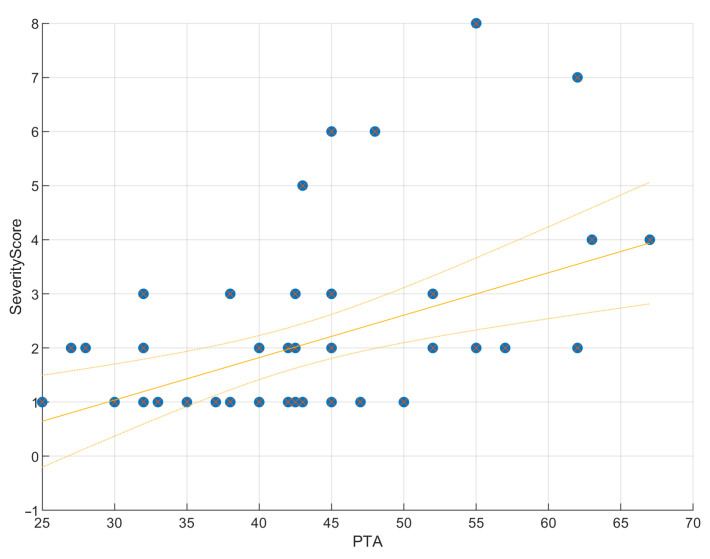
Regression of PTA on severity score. Scatter plot with fitted regression line showing a significant positive correlation (ρ = 0.32, *p* = 0.015). PTA explains 20% of the variance in the Severity Score (R^2^ = 0.20, RMSE = 1.47), indicating that malformation severity is a modest but significant predictor of hearing thresholds. The slope was positive (β = 0.08, SE = 0.02, 95% CI [0.04, 0.12]), suggesting that each unit increase in PTA was associated with an average increase of 0.08 units in Severity Score.

**Figure 4:**
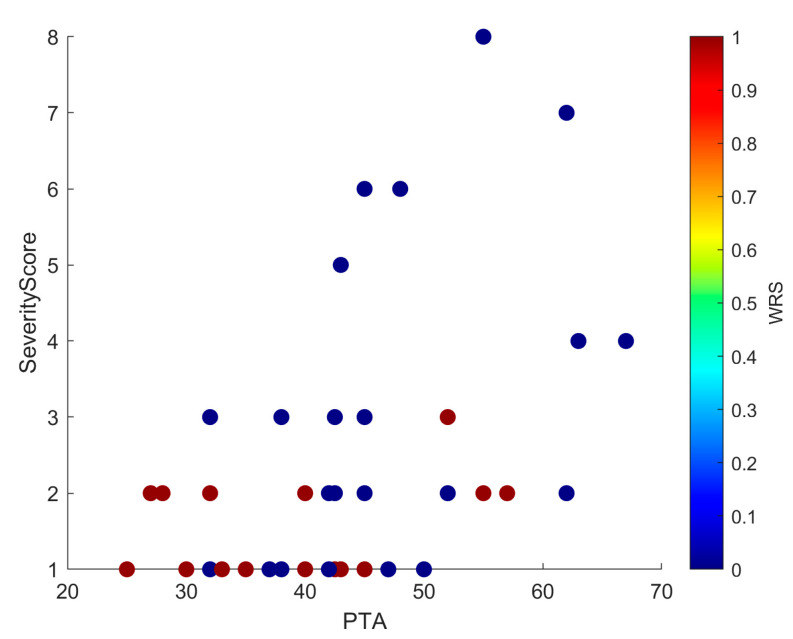
Scatter plot showing PTA and Severity Score, with each point colored by WRS. The distribution mirrors that of Figure 5: low PTA and low Severity Scores cluster with higher WRS, whereas high PTA and high Severity Scores cluster with lower WRS. In addition, from Severity Score ≥ 3 onwards, there is a clear increase in cases with both poorer PTA and reduced WRS, underscoring the negative prognostic effect of greater malformation severity.

**Figure 5:**
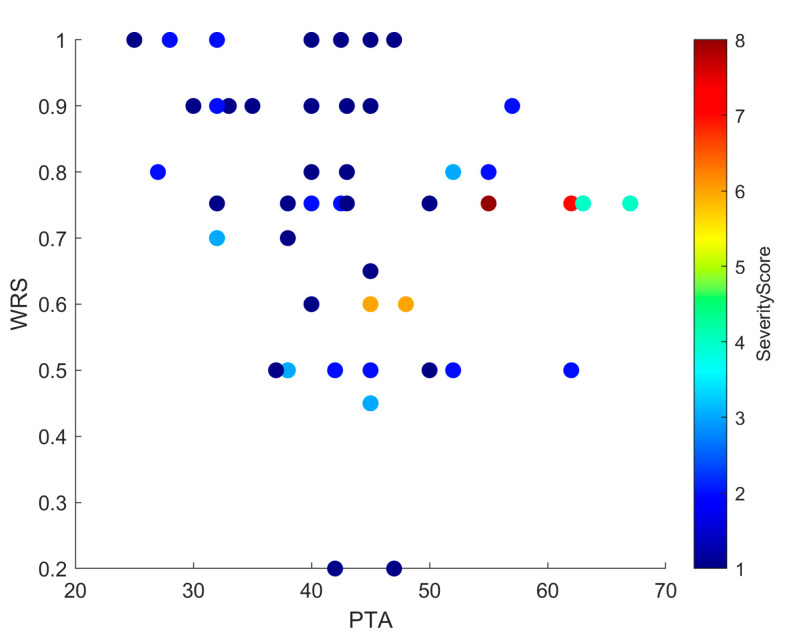
Scatter plot showing the relationship between PTA and WRS, with each point color-coded by Severity Score. Higher Severity Scores tend to cluster with elevated PTA and reduced WRS values, suggesting a negative impact of anatomical complexity on auditory performance. While this trend is evident, some variability across individual cases indicates that other factors may also influence outcomes.

Linear regression confirmed that higher severity scores predicted worse PTA values (R^2^ = 0.20; β = 0.08; SE = 0.02; 95% CI, 0.04–0.12; *p* = 0.015). This positive slope indicated that each unit increase in PTA was associated with an average increase of 0.08 units in Severity Score, supporting anatomical severity as a modest but significant predictor of hearing thresholds (Figure 3). Scatterplots (Figure 4 and Figure 5) further illustrated this trend: ears with low PTA and low Severity Scores generally showed higher WRS, whereas high PTA and high Severity Scores clustered with poorer speech recognition. From Severity Score > 3 onwards, impaired PTA and WRS became more frequent, underscoring the potential prognostic value of this threshold as a marker of increased risk of limited auditory benefit.

#### 3.7.3. Exploratory Multivariate Analyses

**Figure 6:**
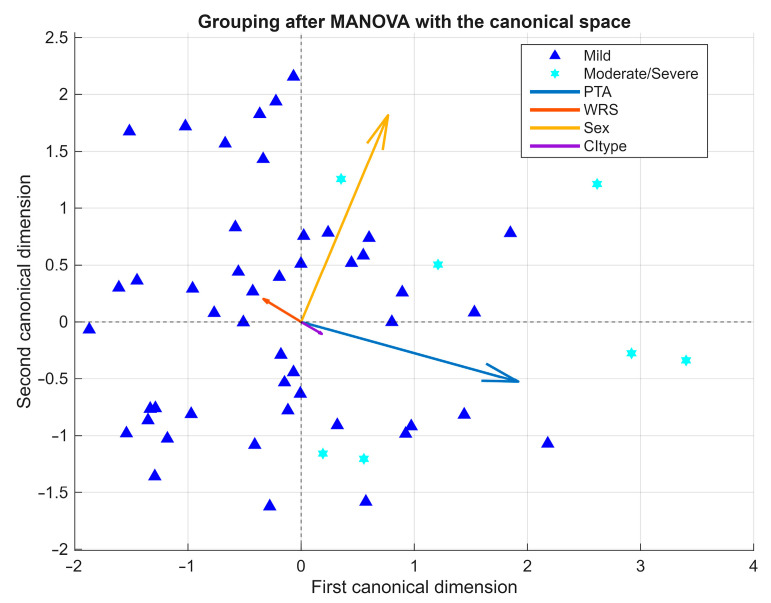
Exploratory MANOVA biplot of severity groups. Biplot of the first two canonical dimensions showing partial separation between mild and moderate/severe malformations. PTA was the main contributor, whereas demographic and device-related factors played a secondary role. Arrows represent variable vectors derived from the correlations between each independent variable and the canonical dimensions, scaled for visual clarity.

As indicated in the previous section, preliminary assumption testing supported the use of MANOVA given its robustness to moderate deviations from normality. An exploratory MANOVA (Figure 6) was performed to evaluate whether combined variable profiles could improve the stratification of malformation severity. The analysis revealed a statistically significant, though moderate, overall group effect in the first canonical dimension (Wilks’ λ = 0.72, χ^2^ = 16.85, df = 4, F ≈ 20.67, *p* = 0.0021). This finding indicates that the joint contribution of the dependent variables accounted for a meaningful portion of between-group variance.

The biplot representation of the first two canonical dimensions showed partial separation between mild and moderate/severe cases. The first canonical dimension was primarily defined by PTA (positive loading) and WRS (negative loading), while the second dimension reflected smaller contributions from CI type, which in this study encompassed both the implant device model (manufacturer and series) and the electrode array design (e.g., Flex 28, Slim Modiolar, Contour Advance). The relatively low Wilks’ Lambda (λ = 0.72) suggests that approximately 28% of the total variance in the dependent variables was explained by group effects, supporting the adequacy of the multivariate approach.

However, the moderate magnitude of the effect and the overlap between clusters—particularly along the first dimension—underscore the exploratory nature of the analysis. Follow-up univariate tests, using partial eta-squared (ηp^2^) as a measure of effect size, revealed a large effect for PTA (ηp^2^ = 0.254), a small effect for Sex (ηp^2^ = 0.102), and negligible effects for WRS (ηp^2^ ≈ 0) and CI type (ηp^2^ = 0.024). Taken together, these results indicate that auditory thresholds were the main contributors to group differentiation, whereas speech perception, sex, and device-related factors (implant and electrode type) played only a minor role.

## 4. Discussion

This retrospective series represents one of the largest single-center descriptions of cochlear implantation in children with IEMs. Our results confirm that CI in this population is generally safe, with a low overall complication rate, and provides meaningful auditory benefit, although performance varies considerably depending on anatomical complexity and cochlear nerve status.

### 4.1. Safety and Complications

Cochlear implantation in children with IEMs poses specific surgical challenges, including the risk of facial nerve anomalies, cerebrospinal fluid (CSF) leak, and incomplete insertion. Careful preoperative imaging and appropriate electrode selection are essential to minimize complications [7,14,15]. CSF gusher is the most frequently reported intraoperative event, especially in common cavity and incomplete partition anomalies, with rates up to 31.6% in common cavity and 28.4% in IP-II [14,16]. In our series, gusher occurred in 3.6% of implanted ears, a lower rate than most reports. The overall complication rate was 10.7% (6/56), with no life-threatening events and all cases successfully managed, comparable or favorable to previous series [15,17]. These findings reinforce that CI in pediatric IEMs is safe.

### 4.2. Auditory and Linguistic Outcomes

CI provided measurable auditory benefit across the spectrum of malformations, consistent with prior studies. [5,15,18]. Ears with EVA and IP-II achieved the best outcomes, with median PTA around 43–45 dB HL and WRS approaching 0.9, whereas more complex malformations such as IP-I, common cavity, and intracochlear ossification were associated with higher thresholds and limited WRS [19]. Posterior labyrinth anomalies showed intermediate and variable results, reflecting frequent overlap with other malformations (Supplemental Table S4).

The exploratory severity score derived from the INCAV classification emerged as a potential prognostic marker. Regression confirmed that higher severity predicted worse PTA (~20% of variance; Figure 3), and scatterplots (Figure 4 and Figure 5) showed that ears with higher scores clustered with elevated PTA and reduced WRS. Outcomes were more frequently impaired from a Severity Score > 3, suggesting a clinically relevant threshold that warrants validation in larger cohorts.

### 4.3. Toward Objective Stratification: INCAV and Severity Scoring

A novel contribution of this study is the application of the INCAV classification, which integrates five anatomical domains into mutually exclusive codes and avoids overlap. The severity score derived from INCAV provided a pragmatic measure of overall anatomical complexity, correlating positively with PTA and negatively with WRS. Exploratory MANOVA showed only partial separation between mild and moderate/severe groups, with PTA and WRS as the main discriminators, while demographic and device-related factors contributed only modestly. Although discriminative power was limited by small subgroup sizes, these findings support the potential of multimodal data approaches to refine prognostic counseling and to standardize reporting across studies.

### 4.4. Cochlear Nerve Status

Cochlear nerve integrity emerged as a critical determinant of outcome. All ears with N2 hypoplasia underwent implantation but showed poorer performance, whereas N3 aplasia cases were not implanted. In the bivariate analysis, nerve deficiency correlated with higher PTA and lower WRS, confirming its role as a negative prognostic marker. Notably, nerve status also correlated strongly with the overall severity score, underscoring its contribution to the composite index of anatomical complexity. This overlap likely explains why the effect of nerve deficiency diminished in multivariate models. These observations are consistent with prior literature [8,20,21] and reinforce the importance of systematically incorporating cochlear nerve assessment into preoperative counseling [22,23].

### 4.5. Risk Factors and Genetic Influences

Perinatal and neonatal risk factors such as NICU stay, low birth weight, meningitis, and CMV infection were relatively frequent but showed only weak or inconsistent associations with auditory outcomes. Similarly, genetic alterations contributed to the heterogeneity of results. Children with SLC26A4 mutations tended to perform relatively well, whereas syndromic cases such as CHARGE or Down syndrome showed poorer thresholds and absent WRS. These observations are consistent with the literature [24,25] (and highlight the importance of integrating genetic and perinatal history into prognostic evaluation, even though anatomical severity remains the dominant determinant of performance.

Certain subgroups require cautious interpretation due to the very small numbers. For instance, the single case with hyperbilirubinemia was implanted at 1 year of age and classified as prelingual, explaining the preserved PTA but WRS of 0%. In the meningitis subgroup, one patient was prelingual at implantation (WRS = 0%), while the other, implanted at 9 years, showed limited speech recognition despite measurable thresholds. These findings illustrate how individual clinical profiles can strongly influence outcomes in rare risk-factor categories and reinforce the need for larger multicenter cohorts to better characterize these subgroups.

### 4.6. Strengths and Limitations

This study has several limitations. First, prelingual children unable to perform speech testing were conservatively coded with WRS = 0%, reflecting the clinical reality at baseline and avoiding overestimation of outcomes. Second, preoperative and postoperative evaluations relied on different tools—mainly electrophysiology (ABR) versus behavioral PTA and WRS—limiting direct longitudinal comparisons. Third, although the number of implanted ears (*n* = 56) is reasonable for a single-center cohort, subgroup analyses were underpowered, reducing the robustness of exploratory models. Fourth, postoperative radiological imaging was not available for all patients, preventing systematic assessment of intracochlear electrode position, which was therefore not included as a study variable. Future research should correlate the INCAV-based severity index with postoperative reconstructions, electrode position, and auditory outcomes. Fifth, in common cavity malformations, the “cochlear nerve present” code (N0) may not fully capture the anatomical reality, underscoring the need to refine the INCAV system. Finally, the proposed severity score remains exploratory and has not yet been externally validated.

Despite these limitations, the study also has important strengths. It represents one of the largest single-center pediatric cohorts of CI in IEMs, with detailed anatomical classification using the novel INCAV system and its derived severity score. The integration of anatomical, genetic, and perinatal factors provides a multidimensional perspective that may enhance prognostic counseling and individualized rehabilitation planning.

## 5. Conclusions

Cochlear implantation in children with inner ear malformations is safe and consistently improves hearing thresholds. Postoperative PTA emerged as the most robust predictor of auditory performance, while the novel severity score derived from the radiological INCAV system correlated with both PTA and WRS. This approach may help identify children at higher risk (scores > 3) and support safer surgical strategies and tailored rehabilitation planning.

## Figures and Tables

**Table 1:** Distribution of inner ear malformations by structure and subtype (*N* = 82).

Structure	Category	*n*	% of Total Malformed Ears
Cochlea	Normal morphology	55	61.4
Cochlea	Incomplete partition type II	18	21.95
Cochlea	Incomplete partition type I	3	3.66
Cochlea	Common cavity	3	3.66
Cochlea	Intracochlear ossification	3	3.66
Posterior labyrinth	Normal	57	64.3
Posterior labyrinth	Dilated semicircular canals	13	15.85
Posterior labyrinth	Absence of semicircular canals	12	14.63
Vestibular aqueduct	Normal	55	61.4
Vestibular aqueduct	Enlarged vestibular aqueduct	27	32.93

Percentages indicate the proportion of ears with each anomaly relative to all malformed ears (*N* = 82). Each anatomical structure—cochlea, posterior labyrinth, and vestibular aqueduct—was analyzed independently; therefore, multiple abnormalities could coexist within the same ear, and the sum of percentages may exceed 100%. Detailed PTA and WRS values for each subtype are available in Supplementary Table S2.

**Table 2:** Distribution of inner ear malformations according to INCAV codes.

INCAV Code	Total Ears *n* (%)	% Implanted Within Malformation	% Non-Implanted Within Malformation	Internal Auditory Canal (I)	Cochlear Nerve (N)	Cochlea (C)	Vestibular Aqueduct (A)	Vestibule (V)
I0 N0 C0 A1 V0	15 (18.3)	13 (86.7)	2 (13.3)	normal	normal	normal	EVA	normal
I0 N0 C0 A0 V1	14 (17.1)	12 (85.7)	2 (14.3)	normal	normal	normal	normal	SSCC malformation
I0 N2 C0 A0 V0	6 (7.3)	6 (100)	0	normal	hypoplasia	normal	normal	normal
I0 N0 C1 A0 V0	5 (6.1)	4 (80)	1 (20)	normal	normal	IP type II	normal	normal
I0 N0 C1 A0 V1	3 (3.7)	2 (66.7)	1 (33.3)	normal	normal	IP type II	normal	SSCC malformation
I2 N0 C5 A0 V5	3 (3.7)	1 (33.3)	2 (66.7)	narrow	normal	CC	normal	CC
I0 N0 C1 A1 V2	3 (3.7)	2 (66.7)	1 (33.3)	normal	normal	IP type II	EVA	dilated
I3 N2 C1 A0 V1	2 (2.4)	1 (50)	1 (50)	atresia	hypoplasia	IP type II	normal	SSCC malformation
I1 N0 C0 A0 V0	2 (2.4)	1 (50)	1 (50)	enlarged	normal	normal	normal	normal
I2 N2 C0 A1 V1	2 (2.4)	2 (100)	0	narrow	hypoplasia	normal	EVA	SSCC malformation
I2 N2 C0 A0 V0	2 (2.4)	2 (100)	0	narrow	hypoplasia	normal	normal	normal
I0 N0 C0 A1 V2	2 (2.4)	1 (50)	1 (50)	normal	normal	normal	EVA	dilated
I0 N0 C1 A1 V0	2 (2.4)	2 (100)	0	normal	normal	IP type II	EVA	normal
I0 N0 C4 A0 V2	2 (2.4)	2 (100)	0	normal	normal	IP type I	normal	dilated
I2 N0 C0 A0 V0	1 (1.2)	1 (100)	0	narrow	normal	normal	normal	normal
I0 N0 C0 A0 V1	1 (1.2)	0	1 (100)	normal	normal	normal	normal	SSCC malformation
I0 N0 C4 A1 V4	1 (1.2)	1 (100)	0	normal	normal	IP type I	EVA	IP type I
I0 N0 C1 A0 V2	1 (1.2)	1 (100)	0	normal	normal	IP type II	normal	dilated
I0 N0 C0 A1 V1	1 (1.2)	1 (100)	0	normal	normal	normal	EVA	SSCC malformation
I0 N0 C0 A0 V2	1 (1.2)	0	1 (100)	normal	normal	normal	normal	dilated
I0 N0 C1 A1 V1	1 (1.2)	1 (100)	0	normal	normal	IP type II	EVA	SSCC malformation

Each INCAV code reflects a unique anatomical profile. In cases of common cavity malformation, the term “cochlear nerve present” refers to the visualization of a cochleovestibular nerve trunk (eighth cranial nerve) within the internal auditory canal, rather than a distinct cochlear branch. EVA = enlarged vestibular aqueduct; IP = incomplete partition; SSCC = semicircular canal malformation; CC = common cavity. Full distribution is available in Supplementary Tables S1 and S2.

**Table 3:** Prevalence of risk factors in the cohort (*N* = 82).

Risk Factor	*n*, (%)	Mean PTA (dB HL)	Mean WRS (%) at 65 dB HL
Genetic alterations	18 (22%)	47	76
SLI	24 (29%)	44	74
NICU Stay	18 (22%)	46	69
Low birth weight	8 (9.8%)	48	65
CMV	6 (7.3%)	37	74
Prematurity	4 (5%)	42	50
Meningitis	3 (3.7%)	42	20
Hyperbilirubinemia	1 (1.2%)	38	0

Percentages refer to the total sample (*N* = 82). Abbreviations: PTA, Pure Tone Average; WRS, Word Recognition Score; SLI, Specific Language Impairment; ASD, Autism Spectrum Disorder; CMV, Cytomegalovirus; NICU, Neonatal Intensive Care Unit. Because not all patients exhibited each risk factor, the total number of cases and percentages do not add up to the overall sample size (82 ears). Each variable was analyzed independently.

**Table 4:** Frequency and auditory outcomes by genetic alteration or comorbid disorder.

Alteration	Frequency	Percentage (%)	PTA Mean (dB HL)	WRS Mean (%) at 65 dB HL
SLC26A4 mutation	4	4.88	44	70
CHARGE syndrome	4	4.88	62	0
GJB2/GJB6 compound heterozygous mutations	2	2.44	38	0
DIABLO (c.592C>T) variant	2	2.44	47	80
Down syndrome	2	2.44	65	0
GJB2 mutation	2	2.44	N/A	N/A
Spinocerebellar ataxia and XYY syndrome	2	2.44	43	50
Epilepsy (unspecified genetic form)	4	2.44	32	0
Usher syndrome type II	2	2.44	32	90

Percentages relative to total ears (*N* = 82). PTA, Pure Tone Average; WRS = word recognition score. N/A = not available. Comorbidities include neurodevelopmental and systemic disorders such as CHARGE syndrome, Down syndrome, epilepsy, and spinocerebellar ataxia.

**Table 5:** Postoperative complications after cochlear implantation (*N* = 56 ears).

Complication	*n* (%)
No complications	50 (89.3 %)
Cerebrospinal fluid gusher	2 (3.6 %)
Reimplantation	2 (3.6 %)
Subperiosteal abscess	1 (1.8 %)
Transient peripheral facial paralysis	1 (1.8 %)

Overall complication rate: 10.7%. All cases resolved without sequelae.

## Data Availability

The data presented in this study are available on reasonable request from the corresponding author. The data are not publicly available due to privacy and ethical restrictions.

## Supplementary Materials

The following supporting information can be downloaded at: https://www.mdpi.com/article/10.3390/jcm14228245/s1. Supplementary Table S1: INCAV Component Coding, Severity Points, and Levels; Supplementary Table S2: Postoperative pure-tone average (PTA) and word recognition score (WRS) by specific malformación subtype. Supplementary Table S3: Distribution of implanted ears with inner ear malformations and electrode models (*n* = 56). Supplementary Table S4: Distribution of Inner Ear Malformations by INCAV Code, Implantation Status, and Severity Score. Supplementary Figure S1: Representative intraoperative, postoperative, and 3D imaging examples related to cochlear implantation in patients with inner ear malformations.

## Abbreviations

The following abbreviations are used in this manuscript:

Abbreviation	Definition
ABR	Auditory Brainstem Response
CAP	Categories of Auditory Performance
CI	Cochlear Implantation
CMV	Cytomegalovirus
CSF	Cerebrospinal Fluid
EVA	Enlarged Vestibular Aqueduct
IEM	Inner Ear Malformation
INCAV	Internal Auditory Canal, Nerve, Cochlea, Aqueduct, Vestibule (classification system)
IP	Incomplete Partition
IP-I	Incomplete Partition Type I
IP-II	Incomplete Partition Type II
LBW	Low Birth Weight
MANOVA	Multivariate Analysis of Variance
NICU	Neonatal Intensive Care Unit
NAMES	Nottingham Auditory Milestones
PTA	Pure-Tone Average
SE	Standard Error
SLI	Specific Language Impairment
WRS	Word Recognition Score

## References

[1] Sennaroglu L., Saatci I. (2002). A New Classification for Cochleovestibular Malformations. Laryngoscope.

[2] Daneshi A., Farhadi M., Ajalloueyan M., Rajati M., Hashemi S.B., Ghasemi M.M., Emamdjomeh H., Asghari A., Mohseni M., Mohebbi S. (2020). Cochlear Implantation in Children with Inner Ear Malformation: A Multicenter Study on Auditory Performance and Speech Production Outcomes. Int. J. Pediatr. Otorhinolaryngol..

[3] Manrique M., Ramos Á., De Paula Vernetta C., Gil-Carcedo E., Lassaletta L., Sanchez-Cuadrado I., Espinosa J.M., Batuecas Á., Cenjor C., Lavilla M.J. (2019). Guía clínica sobre implantes cocleares. Acta Otorrinolaringológica Española.

[4] Jackler R.K., Luxfor W.M., House W.F. (1987). Congenital Malformations of the Inner Ear: A Classification Based on Embryogenesis. Laryngoscope.

[5] Sennaroğlu L., Bajin M.D. (2017). Classification and Current Management of Inner Ear Malformations. Balk. Med. J..

[6] Adibelli Z.H., Isayeva L., Koc A.M., Catli T., Adibelli H., Olgun L. (2017). The New Classification System for Inner Ear Malformations: The INCAV System. Acta Oto-Laryngol..

[7] Pakdaman M.N., Herrmann B.S., Curtin H.D., Van Beek-King J., Lee D.J. (2012). Cochlear Implantation in Children with Anomalous Cochleovestibular Anatomy: A Systematic Review. Otolaryngol. Head Neck Surg..

[8] Wu C., Chen Y., Chen P., Hsu C. (2005). Common Clinical Features of Children with Enlarged Vestibular Aqueduct and Mondini Dysplasia. Laryngoscope.

[9] Alahmadi A., Abdelsamad Y., Salamah M., Alenzi S., Badr K.M., Alghamdi S., Alsanosi A. (2022). Cochlear Implantation in Adults and Pediatrics with Enlarged Vestibular Aqueduct: A Systematic Review on the Surgical Findings and Patients’ Performance. Eur. Arch. Otorhinolaryngol..

[10] Forli F., Lazzerini F., Auletta G., Bruschini L., Berrettini S. (2020). Enlarged Vestibular Aqueduct and Mondini Malformation: Audiological, Clinical, Radiologic and Genetic Features. Eur. Arch. Oto-Rhino-Laryngol..

[11] Archbold S., Lutman M.E., Marshall D.H. (1995). Categories of Auditory Performance. Ann. Otol. Rhinol. Laryngol. Suppl..

[12] Datta G., Kitterick P.T., Ramirez-Inscoe J. (2018). Development and Validation of the Nottingham Auditory Milestones (NAMES) Profile for Deaf Children under 2 Years Old, Using Cochlear Implants. Cochlear Implant. Int..

[13] De Cardenas M., Marrero Aguiar V. (1994). Cuaderno de Logoaudiometría.

[14] Farhood Z., Nguyen S.A., Miller S.C., Holcomb M.A., Meyer T.A., Rizk A.H.G. (2017). Cochlear Implantation in Inner Ear Malformations: Systematic Review of Speech Perception Outcomes and Intraoperative Findings. Otolaryngol. Head Neck Surg..

[15] Bille J., Fink-Jensen V., Ovesen T. (2015). Outcome of Cochlear Implantation in Children with Cochlear Malformations. Eur. Arch. Otorhinolaryngol..

[16] Demir B., Cesur S., Sahin A., Binnetoglu A., Ciprut A., Batman C. (2019). Outcomes of Cochlear Implantation in Children with Inner Ear Malformations. Eur. Arch. Otorhinolaryngol..

[17] Sbeih F., Bouzaher M.H., Appachi S., Schwartz S., Cohen M.S., Carvalho D., Yoon P., Liu Y.-C.C., Anne S. (2022). Safety of Cochlear Implantation in Children 12 Months or Younger: Systematic Review and Meta-Analysis. Otolaryngol. Head Neck Surg..

[18] Papsin B.C. (2005). Cochlear implantation in children with anomalous cochleovestibular anatomy. Laryngoscope.

[19] Liu X., Chao X., Wang R., Luo J., Wang M., Li J., Hu F., Xu L. (2025). Long-Term Auditory and Speech Outcomes of Cochlear Implantation in Children with IP-I Malformation. Laryngoscope.

[20] Sennaroglu L. (2010). Cochlear Implantation in Inner Ear Malformations—A Review Article. Cochlear Implant. Int..

[21] Birman C.S., Powell H.R., Gibson W.P., Elliott E.J. (2016). Cochlear implant outcomes in cochlear nerve aplasia and hypoplasia. Otol. Neurotol..

[22] Isaiah A., Lee D., Lenes-Voit F., Sweeney M., Kutz W., Isaacson B., Roland P., Lee K.H. (2017). Clinical Outcomes Following Cochlear Implantation in Children with Inner Ear Anomalies. Int. J. Pediatr. Otorhinolaryngol..

[23] Young N.M., Kim F.M., Ryan M.E., Tournis E., Yaras S. (2012). Pediatric cochlear implantation of children with eighth nerve deficiency. Int. J. Pediatr. Otorhinolaryngol..

[24] Shearer A.E., Hildebrand M.S., Schaefer A.M., Smith R.J., Adam M.P., Feldman J., Mirzaa G.M., Pagon R.A., Wallace S.E., Amemiya A. (1993). Genetic Hearing Loss Overview. GeneReviews®.

[25] Lazzarotto T., Blázquez-Gamero D., Delforge M.-L., Foulon I., Luck S., Modrow S., Leruez-Ville M. (2020). Congenital Cytomegalovirus Infection: A Narrative Review of the Issues in Screening and Management from a Panel of European Experts. Front. Pediatr..

