# Supplementary material for: Cochlear Implantation in Children with Inner Ear Malformations: Auditory Outcomes, Safety and the Role of Anatomical Severity
Source: J Clin Med. 2025 Nov 20;14(22):8245. doi: 10.3390/jcm14228245 (PMC12653052; doi:10.3390/jcm14228245)
Supplement: Supplementary file 1 [file jcm-14-08245-s001.zip › jcm-3931757-supplementary.pdf]

Supplementary Methods: INCAV-Derived Severity Score

Inner ear malformations (IEMs) were independently identified by two otologists and two radiologists using high-resolution CT and MRI. Each case was classified with the INCAV system (Adibelli et al. 2017), which evaluates five domains: internal auditory canal (I), cochlear nerve (N), cochlea (C), vestibular aqueduct (A), and vestibule (V). Each ear received a unique INCAV code to avoid subgroup overlap.

During data analysis, conventional classifications proved difficult to apply consistently, as many ears displayed more than one anomaly and patients were duplicated across subgroups. To overcome this limitation, we developed an exploratory **severity score** derived from the INCAV domains. Each component was assigned an ordinal value from 0 (normal) to 3 (severe defect), and the sum of all domains produced a global severity index ranging from 0 to 13 points.

Ears were stratified into three categories:

- **Mild (1–3 points):** typically, isolated anomalies (e.g., enlarged vestibular aqueduct or semicircular canal malformations), generally compatible with favourable implantability.
- **Moderate (4–7 points):** more complex but still implantable patterns (e.g., incomplete partition type II, combined vestibular–cochlear anomalies).
- **Severe (≥8 points):** multi-domain involvement (e.g., common cavity, incomplete partition type I, cochlear nerve hypoplasia/aplasia).

This stratification avoided duplication, provided a pragmatic measure of overall anatomical complexity, and reflected our observation that higher INCAV values seem to correlate with poorer auditory outcomes.

Supplementary Table S1: INCAV Component Coding, Severity Points, and Levels.

Legend: This table summarizes the coding of each INCAV component—internal auditory canal (I), cochlear nerve (N), cochlea (C), vestibular aqueduct (A), and vestibule (V)—with the corresponding anatomical description, severity points (0–3), and severity level (normal, mild, moderate, severe). The scoring system was used to derive the global severity index for each ear, with a possible range of 0–13 points. Higher scores indicate greater anatomical complexity and severity.

Code	Structure	Description	Severity Points	Severity Level
**I0**	Internal auditory canal	Normal	0	Normal
**I1**	Internal auditory canal	Enlarged	1	Mild defect
**I2**	Internal auditory canal	Narrow	2	Moderate defect
**I3**	Internal auditory canal	Atresia	3	Severe defect
**N0**	Cochlear nerve	Normal	0	Normal
**N1**	Cochlear nerve	Thick/abnormal (MRI or CT)	1	Mild defect
**N2**	Cochlear nerve	Hypoplasia	2	Moderate defect
**N3**	Cochlear nerve	Aplasia	3	Severe defect
**C0**	Cochlea	Normal cochlea	0	Normal
**C1**	Cochlea	Incomplete partition type II (Mondini)	1	Mild defect
**C2**	Cochlea	Incomplete partition type III	1	Mild defect
**C3**	Cochlea	Cochlear hypoplasia	2	Moderate defect
**C4**	Cochlea	Incomplete partition type I / cystic cochleovestibular anomaly	2	Moderate defect
**C5**	Cochlea	Common cavity	3	Severe defect
**C6**	Cochlea	Cochlear aplasia	3	Severe defect
**A0**	Vestibular aqueduct	Normal	0	Normal
**A1**	Vestibular aqueduct	Enlarged vestibular aqueduct	1	Mild defect
**V0**	Vestibule	Normal	0	Normal
**V1**	Vestibule	Semicircular canal malformation	1	Mild defect
**V2**	Vestibule	Dilated vestibule	1	Mild defect
**V3**	Vestibule	Vestibular hypoplasia	2	Moderate defect
**V4**	Vestibule	Incomplete partition type I / cystic cochleovestibular anomaly	2	Moderate defect
**V5**	Vestibule	Common cavity	3	Severe defect
**V6**	Vestibule	Cochlear aplasia	3	Severe defect

Supplementary Table S2. Postoperative pure-tone average (PTA) and word recognition score (WRS) by specific malformación subtype.

Legend:
Median [IQR] values are shown for ears with available postoperative data at one year. PTA is expressed in dB, WRS as percentage correct (0%-100%). Categories with very small sample sizes should be interpreted with caution.

Structure	Category	n (PTA)	PTA Median [IQR]	n (WRS)	WRS Median [IQR]
**Cochlea**	Incomplete partition type II	14	45 [18.8]	12	90% [12,5%]
**Cochlea**	Incomplete partition type I	3	50 [5.5]	2	60% [10%]
**Cochlea**	Common cavity	1	55 [0.0]	1	0%
**Cochlea**	Intracochlear ossification	3	42 [5.0]	3	20% [15%]
**Posterior labyrinth**	Dilated semicircular canals	10	38.5 [14.5]	6	60% [30%]
**Posterior labyrinth**	Absence of semicircular canals	7	43 [6.5]	5	70% [40%]
**Vestibular aqueduct**	Enlarged vestibular aqueduct	24	43 [5.5]	19	90% [32,5%]

Supplementary Table S3. Distribution of implanted ears with inner ear malformations and electrode models (n = 56).

Device	Common Cavity	Normal Morphology	Intracochlear Ossification	Incomplete Partition Type I	Incomplete Partition Type II	Total
**AB HiRes 90K (Advanced Bionics)**	0	3	0	0	0	3
**AB HiFocus Mid-Scala (Advanced Bionics)**	0	4	0	0	0	4
**AB Ultra 3D Mid-Scala (Advanced Bionics)**	0	6	0	0	0	6
**CI632 Slim Modiolar (Cochlear)**	0	7	0	0	1	8
**Concerto MI1000 Compress (MED-EL)**	0	0	0	0	1	1
**Concerto MI1000 Flex 28 (MED-EL)**	0	1	**1**	0	1	3
**Nucleus CI512 Contour Advance (Cochlear)**	0	4	**1**	0	1	6
**Nucleus CI622 (Cochlear)**	0	2	0	0	0	2
**Synchrony 2 MI1250 Flex 28 (MED-EL)**	0	7	0	0	3	10
**Synchrony 2 MI1250 Form 19 (MED-EL)**	**1**	0	0	0	0	1
**Synchrony 2 MI1250 Form 24 (MED-EL)**	0	0	0	0	3	3
**Synchrony MI1200 Flex 28 (MED-EL)**	0	0	0	**1**	0	1
**Synchrony MI1200 Form 19 (MED-EL)**	0	0	0	**2**	1	3
**Sonata TI100 (MED-EL)**	0	2	**1**	0	2	5
**Total**	1	36	3	3	13	56

Abbreviations: n, number of implanted ears; MED-EL, Advanced Bionics (AB), and Cochlear (CI) denote implant manufacturers. Cochlear malformations include incomplete partition types I–II, common cavity, and intracochlear ossification. The electrode array selection was primarily guided by the cochlear malformation. Posterior labyrinth anomalies played a secondary role, explaining the presence of some ears with normal cochlear morphology but minor posterior alterations less relevant to electrode choice.

Supplementary Table S4. Distribution of Inner Ear Malformations by INCAV Code, Implantation Status, and Severity Score

This table details each unique INCAV code identified in the cohort, showing the total number and percentage of ears, the proportion of implanted and non-implanted ears, and their corresponding Severity Score (0–13) and Severity Group (mild, moderate, severe). Severity scores were calculated by summing the points assigned to each INCAV domain, as described in Supplementary Table 1.

INCAV code	Total ears N (%)	% implanted within malformation	% non-implanted within malformation	Severity Score (0–13)	Severity Group
**I0 N0 C0 A1 V0**	15 (18,3)	13 (86,7)	2 (13,3)	1	Mild
**I0 N0 C0 A0 V1**	14 (17,1)	12 (85,7)	2 (14,3)	1	Mild
**I0 N2 C0 A0 V0**	6 (7,3)	6 (100)	0	2	Mild
**I0 N0 C1 A0 V0**	5 (6,1)	4 (80)	1 (20)	1	Mild
**I0 N0 C1 A0 V1**	3 (3,7)	2 (66,7)	1 (33,3)	2	Mild
**I2 N0 C5 A0 V5**	3 (3,7)	1 (33,3)	2 (66,7)	8	Severe
**I0 N0 C1 A1 V2**	3 (3,7)	2 (66,7)	1 (33,3)	3	Mild
**I3 N2 C1 A0 V1**	2 (2,4)	1 (50)	1 (50)	7	Moderate
**I1 N0 C0 A0 V0**	2 (2,4)	1 (50)	1 (50)	1	Mild
**I2 N2 C0 A1 V1**	2 (2,4)	2 (100)	0	6	Moderate
**I2 N2 C0 A0 V0**	2 (2,4)	2 (100)	0	4	Moderate
**I0 N0 C0 A1 V2**	2 (2,4)	1 (50)	1 (50)	2	Mild
**I0 N0 C1 A1 V0**	2 (2,4)	2 (100)	0	2	Mild
**I0 N0 C4 A0 V2**	2 (2,4)	2 (100)	0	3	Mild
**I2 N0 C0 A0 V0**	1 (1,2)	1 (100)	0	2	Mild
**I0 N0 C0 A0 V1**	1 (1,2)	0	1 (100)	1	Mild
**I0 N0 C4 A1 V4**	1 (1,2)	1 (100)	0	5	Moderate
**I0 N0 C1 A0 V2**	1 (1,2)	1 (100)	0	2	Mild
**I0 N0 C0 A1 V1**	1 (1,2)	1 (100)	0	2	Mild
**I0 N0 C0 A0 V2**	1 (1,2)	0	1 (100)	1	Mild
**I0 N0 C1 A1 V1**	1 (1,2)	1 (100)	0	3	Mild

Supplementary Figure S1.

Representative intraoperative, postoperative, and 3D imaging examples related to cochlear implantation in patients with inner ear malformations. Four illustrative cases are presented: two incomplete partition type I (B), one common cavity, and one incomplete partition type II (Mondini) malformation.

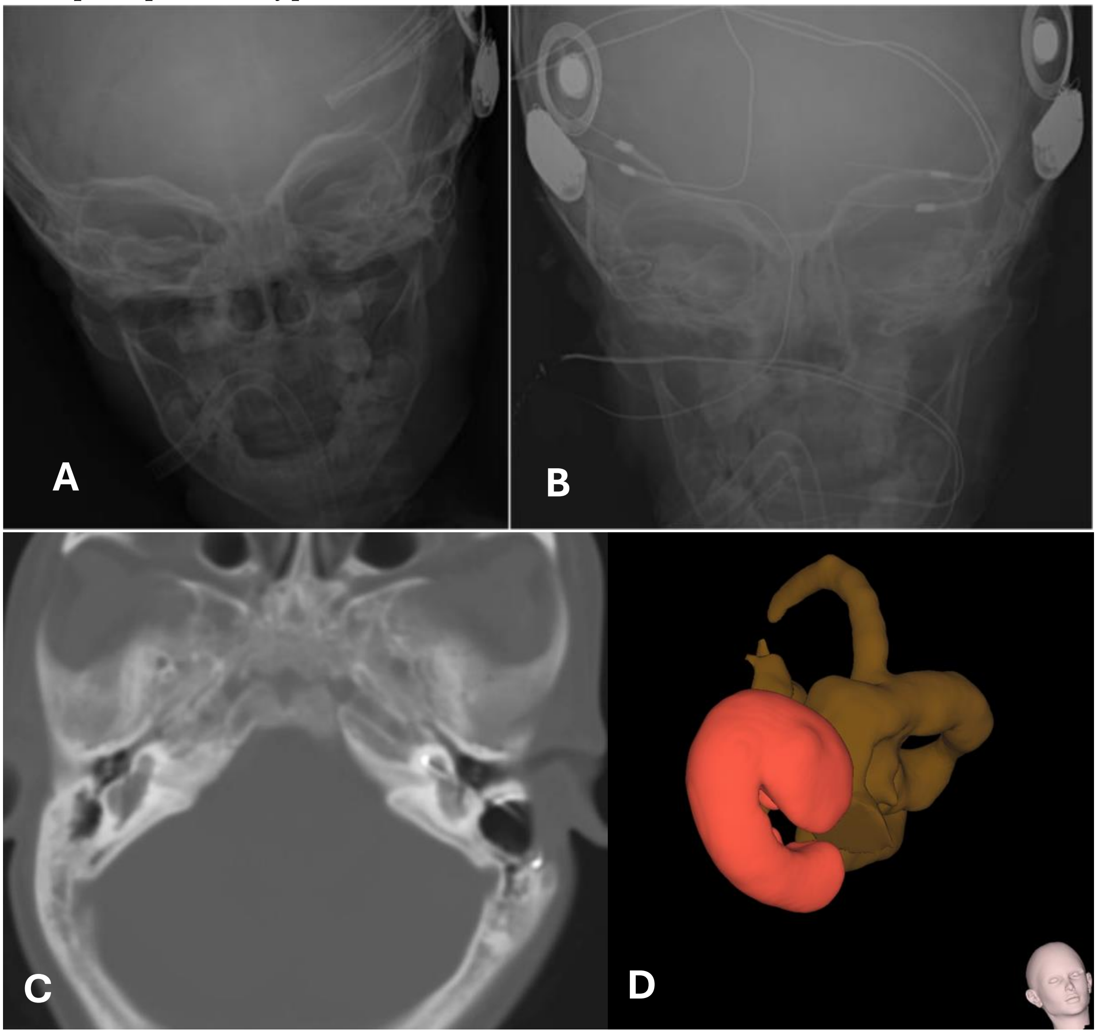

(A) Left ear, intraoperative radiograph of a patient with incomplete partition type I implanted with a Synchrony MI1200 Flex 28 (MED-EL, Innsbruck, Austria) electrode. A perilymph gusher occurred during surgery and was successfully sealed using muscle tissue and fibrin glue (Tissucol).
(B) Bilaterally implanted patient with incomplete partition type I using Synchrony MI1200 Form 19 (MED-EL, Innsbruck, Austria) electrodes. The shorter and stiffer design with SEAL technology allowed controlled insertion and minimized cerebrospinal fluid leakage.
(C) Left ear, postoperative CT scan of a common cavity malformation, characterized by fusion of the cochlea and vestibule into a single cystic cavity, implanted with a Synchrony 2 MI1250 Form 19 (MED-EL, Innsbruck, Austria) electrode.
(D) Three-dimensional reconstruction obtained with OTOPLAN® software (CASCINATION AG, Version 3.0; MED-EL, Innsbruck, Austria) illustrating an incomplete partition type II (Mondini) malformation with 1.5 cochlear turns, incomplete interscalar septum, and partial absence of the superior semicircular canal.
